# Developments in Drug Addiction During COVID-19—An Austrian Perspective Based on a Clinical Sample

**DOI:** 10.3389/fpsyt.2020.602033

**Published:** 2020-11-27

**Authors:** Isabella Fuchs-Leitner, Kurosch Yazdi, Nikolas W. Gerstgrasser, Jan Rosenleitner

**Affiliations:** ^1^Department of Psychiatry - Specialization Addiction Medicine, Kepler University Hospital, Linz, Austria; ^2^Faculty of Medicine, Johannes Kepler University Linz, Linz, Austria

**Keywords:** COVID-19, drug addiction, consumption pattern, illicit drug market, opioid substitution therapy (OST)

## Abstract

Concerns about the negative consequences of the COVID-19 pandemic on people with substance use disorder (SUD) were raised by experts in the field around the world. Here we provide an Austrian perspective, discussing the impact of the pandemic on help-seeking patient with drug use disorder during the initial stage of the pandemic. Our perspectives are based on the situation as perceived at our clinical facility, and supported by original data collected from a small clinical sample of patients with drug use disorder (*N* = 32). The viewpoints and related descriptive data include the perceived individual impact of COVID-19, as well as various aspects of drug use behavior and the Austrian drug market before and after the onset of the pandemic. The consequences for a subgroup of patients in opioid substitution treatment (*N* = 24) are discussed. Surprisingly and in contrast to anticipated developments, we had the impression of a rather stable situation in Austria, at least at this early stage of the pandemic. The immediate impact of COVID-19 on these help-seeking patients with high levels of drug dependency seemed less severe than anticipated so far. Importantly, this observation might be a short-term effect for this already fragile group and careful monitoring of further developments as well as preparation of long-term strategies are advised. In general, problematic drug use is associated with many health risk factors and finding appropriate long-term health care strategies has to remain a top priority facing the pandemic. Our perspectives are restricted to observations from help-seeking patients at our clinic, and no conclusions for the general population can be directly drawn.

## Introduction

Experts around the world have clearly articulated their concerns about the impact and consequences of the COVID-19 pandemic on the mental health. The impact of COVID-19 might be particularly challenging for vulnerable populations (1) including people suffering from substance use disorder (SUD) (2). The reciprocal impact between Covid-19 and SUD have been described, categorized in spread of disease, risk of infection, increased severity of COVID-19 symptoms, psychological stress, and reduced access to addiction treatment services (3). Reports from different countries suggest reduced availability of illicit and prescribed drugs, altered consumption patterns, higher probability of relapse, and even elevated risk of deadly overdose without opportunity for rescue due to social distancing and isolation (4–8). All this is seen as a result of government control strategies and border closures, leading to interruptions in illegal drug supply, self-manufacturing of substances, changes in quality and strength of those substances, poor access to health services, psychological stress due to isolation, worries about employment, and personal financial situation and even suicide (9–13).

As a direct consequence, people who use drugs (PWUD) are at higher risk of COVID-19 from a physiological perspective (4). Preexisting conditions regarding the respiratory system from inhalation drugs, damaging effects of drugs on the cardiovascular system and an overall worse health condition further increase the risk of mortality associated with COVID-19 (10). In fact, mortality in the population with OUD appears to be higher than in the general population (6). From a psychological perspective, recent literature indicate a serious impact of COVID-19 on the feelings, thoughts and behavior of patients with substance addiction (14, 15). The current pandemic can lead to indirect consequences on PWUD, as additional stressors on mental health conditions could trigger relapses (5). Direct and indirect consequences can even grow more acute for PWUD given the poor access to health services (9). For patients in opioid substitution treatment (OST), misuse and diversion of OST medicine can result in many negative effects on health, including risks from injecting behavior and overdose, and these problems have been discussed long before the COVID-19 crisis (16). Furthermore, progress in recovery might be at risk and the indirect impact on the whole society ranges from economic costs of untreated opioid dependence to drug-related criminal behavior (17). In the context of COVID-19, experts warn about fatal opioid poisoning due to increased medication diversion (10). People in OST already experience vulnerabilities in their medical, mental, and social health (13), making the COVID-19 pandemic as potential source of additional distress especially challenging. Providing stable OST services for this clinical population is therefore advised to remain a priority (13).

**Figure 1:**
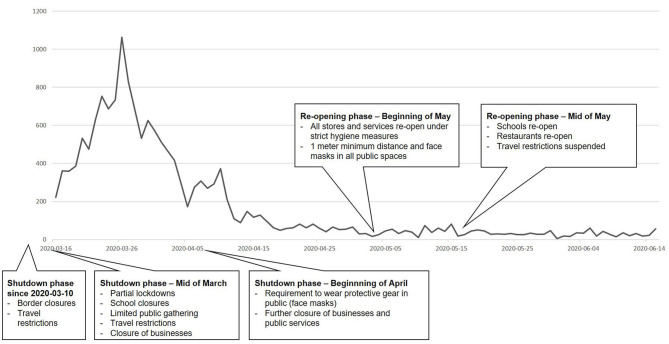
COVID-19 in Austria between mid of March and mid of June 2020: confirmed cases per day and related government measures during the shutdown and re-opening phases.^1^

Regarding the initial stage of the pandemic in Austria, cases of confirmed COVID-19 (total population of 8.859 Million) are displayed in Figure 1 between March and June 2020. Government measures for health care systems included reduction of face-to-face contacts, postponement of non-urgent procedures and major restrictions for outpatient clinics. For most patients in OST, less strict regulations were applied for medication prescription (extension from 1–2 months) and dispensation (from daily to weekly). In sum, Austria adopted early and aggressive control strategies (18). Development of COVID-19 incidents and mortality was comparable to other European countries like Germany or Switzerland at this stage of the pandemic.

Closely looking at the situation reported by health care systems of other countries, we feared a major impact on our drug addicted patients, whether in OST or not. As the largest addiction care facility in our province (Upper Austria) we prepared for different scenarios including an onrush of patients suffering from withdrawal due to reduced availability of illicit drugs or relapse of former patients, loss of contact in ongoing OST due to restricted access to our outpatient clinic, severe intoxications due to altered consumption patterns, etc. As an attempt to quantify the impact of COVID-19 pandemic on our patients, we added specific questions to our routine anamneses for later analysis.

Data is presented in a descriptive manner additionally to our perspectives in the following sections (for more details see tables in the Supplementary Material). Our sample consisted of 32 patients (27 male, 5 female; mean age = 28.8 years), who sought treatment for drug addiction at our clinical facility. Data collection was conducted in accordance to the Declaration of Helsinki and approved by the local ethics committee. Current drug consumption was evaluated by the first four items of the “Drug Used Identification Test” (19) [DUDIT-C (20) with a total score ranging between 0 and 16], whereas subjective craving was indicated by the patients on a 5-point Likert-scale ranging from 0 (no craving) to 4 (strong craving). Additionally, various aspects of drug consumption patterns and drug supply (e.g., availability and prices) were evaluated, and perceived changed due to COVID-19 were documented. For patients in substitution treatment (*N* = 24) misuse and concomitant use of other drugs were assessed. Data collection started 1 month after the onset of the COVID-19 crisis in Austria, determined by the first official Austrian government measures mid of March 2020. Data was collected for 2 months (mid of April until mid of June 2020). Please note that this original data supports our personal perspectives, but that our views are based on our general perception of the situation in a clinical setting at the beginning of the pandemic.

## Perspectives

### Individual Impact of COVID-19 on Drug Use Behavior

Our overall impression at our clinical facility was that patients were less affected by the pandemic than anticipated. In this clinical setting often mostly highly addicted patient are treated, which is also reflected by high levels of drug dependency in our sample (mean DUDIT-C score = 9.9; mean craving = 2.3, correlation coefficient Spearman's *rho* = 0.43, *p* = 0.015). The impact of COVID-19 on personal life was categorized into physiological, psychological, economic, social, and other aspects, and indicated by the patients as either absent or present (see Table 1).

**Table 1:** Individual impact of COVID-19 on different areas of life: Physiological, psychological, economic, social, and other factors are displayed with respective examples, total numbers and percentages (*N* = 32; multiple references were possible).

**COVID-19 factors**	**Examples**	**Total number**	**Percent**
Physiological	Health problems; access to health care	10	31.3
Psychological	Anxiety; depression; anger	20	62.5
Economic	Financial troubles; job loss	9	28.1
Social	Isolation; visitor restrictions	16	50.0
Others	Drug acquisition	10	31.3

At the beginning of the pandemic, especially psychological and social aspects seemed to affect the personal life. Among our sample, struggling with anxiety, fear, and isolation was reported, but no direct association with factors due to COVID-19 could be observed for levels of craving or drug dependency.

From our point of view, drug consumption patterns seemed hardly affected by the COVID-19 pandemic among the patients in Austria at the initial stage. Preferred drugs and consumption forms appeared to be unaltered by COVID-19, which was also reflected in our sample. (All consumed drugs and substances, as well as those indicated as “preferred drug” are displayed in Supplementary Table 1). None of our patients indicated a change in their preferred drug, nor how they consumed it (i.e., inhalative, intravenous, oral etc.) before and after the onset of COVID-19. Related government measures like physical distancing resulted in reduced contact only for the minority of our participants, in terms of consuming alone instead of in groups or only in private spaces. Furthermore, we found a wide range of consumed illicit drugs in our sample, with many reporting regular consumption of more than one substance or drug. The unaltered pattern of consumption is also tightly connected to a stable drug availability at the illicit drug market.

Our impression is that this group of patients was struggling with many aspects brought along by the pandemic. These aspects include high levels of unemployment, financial instability, health problems, social isolation, and psychological stress. This might be a reason why the observed direct impact of COVID-19 on drug use behavior seems less severe at this initial stage, but can result in fatal long-term effects, if no specific treatment for this group is provided. Therefore, a special emphasis on this already deprived population is of utmost importance to avoid a further downward spiral, and enabling access to psychological support and therapy is essential during the next phases of the pandemic.

### Developments at the Illegal Drug Market in Austria

Developments of the illegal drug market were deflected by participants' information regarding source, pricing and quality of illicit drugs, as well as other aspects of drug supply and potential changes due to COVID-19 (see Supplementary Table 2). The way of receiving drugs (active: having to leave the house; passive: getting drugs delivered) did not change for any of our participants, even though government measures included movement restrictions.

Only 16% of our sample reported changes in their usual source of drugs due to COVID-19. In terms of availability and pricing, only a small proportion reported increased difficulties (from 9% before to 22% after COVID-19) in obtaining certain substances. An increase in pricing was indicated by 20% of our patients and reported for heroin, cannabis, and methamphetamine. The majority of our patients (81%) judged the quality of the consumed substances unaffected by the pandemic. Stockpiling of drugs due to concerns about future availability was not observed in our study, and expected disruption in drug availability (21) could rarely be observed.

Overall, the situation at the Austrian drug market seemed stable at the initial stage of COVID-19. This might be related to the fact that in Austria COVID-19 incidence (i.e., confirmed cases relative to the size of the population) was lower compared to many other European countries and also worldwide so far (18). This overall impression of stability is in line with expert opinions on drug retail prices and availability at the consumer level reported for Austria (EMCDDA: European Monitoring Center for Drugs and Drug Addiction) (22). According to this report, this stability on the drug market could also be observed for Czechia, Hungary, Netherlands, and Sweden, whereas changes were perceived for countries heavily impacted by COVID-19 like France or Spain (22). In contrast, the EMCDDA expected a decline in drug use during the first 3 months of the pandemic (as summarized in their related trendspotter briefing) (23). While this might be true for other countries or in other populations with lower levels of drug dependency like recreational drug users or social substance use, our results did not confirm this anticipation. This observation is restricted to the initial stage of the pandemic, but we do not expect a long-term diminution for this specific population due to lack of drug availability or increase in pricing. In other words, drug addiction will not disappear due to outer circumstances, and again, stability in treatment and therapy is strongly advised.

### Patients in Opioid Substitution Treatment: Misuse and Concomitant Use

Misuse and concomitant use among patients in OST appeared to be a prevalent problem, even before the pandemic. We anticipated that the less restricted access to substitution medicine might lead more patients to use OST medication divergent from its purpose. From our point of view, it would also have been possible that disruption in illicit drug supply might lead patients to less concomitant use of other drugs. Another expectation was that new patients were prone to start OST due to a potential lack of availability in opioids at the illicit drug markets. All of these anticipations were not confirmed by our observations.

Among our sample of patients in OST misuse and diversion were found to be very common. Patients in OST (*N* = 24) were evaluated as a subsample regarding misuse and diversion of OST medication, as well as concomitant use of other illicit drugs (see Supplementary Table 3). In Austria pharmacological treatment in OST includes buprenorphine, buprenorphine/naloxone, methadone, levomethadone, and retarded morphine. In our sample 79% reported concomitant use of other illicit substances. Misuse (e.g., injecting or snorting) of the prescribed oral OST medication was indicated by 50%, with estimates on how often they used their OST medication divergent from the prescription ranging from 20 to 100% (mean 92.5%). In respect to diversion, 16.7% reported additional consumption of unprescribed OST medication. We further asked all participants (*N* = 32) for their judgment on the frequency of misuse and diversion of OST medication in their social environment. Fifty-six percentage indicated misuse of OST medication by others, with estimated misuse frequencies ranging between 20 and 100% (mean = 79.4%). Again, no changes between before and after the onset of COVID-19 were observed. The remaining 44% of participants did not provide an answer.

Importantly, no changes in consumption patterns related to OST due to COVID-19 were indicated at all. In Austria, access to health care providers (1) was less affected than the situation required in heavily impacted countries like Italy, Spain, or France. Essentially, regulations regarding prescriptions for OST medication were temporarily eased to ensure maintenance of therapy despite the lock down. It is widely acknowledged that misuse and regular concomitant use of illicit drugs in addition to prescribed OST medication is highly prevalent among these patients (17). In our opinion, the impression that the less rigid OST supply policies had no direct impact on these problematic topics, could only be a short-term effect and the situation can get out of hand rapidly. From our perspective, during lock-down only the main pharmacological supply of these patients was enabled, while long-term treatment including psychological and psychiatric support was nearly impossible due to restricted access to all outpatient clinics. For the future, it is important to provide patients suffering from addictive disorders with all possible resources in order to maintain a high standard in addiction care practice, including use of telehealth and adopting proactive policies (3). In this context, we strongly recommend the EMCDDA's conclusion that developments in the area of PWUD due to COVID-19 should be closely monitored in respect to potential risky and hazardous patterns of use (23).

### Risk of Overdose Due to COVID-19

Many factors that are brought along by the predominant COVID-19 crisis lead to an anticipated increase in overdoses and fatal outcomes, including disruption in drug supply and social distancing (13). In Austria these risk factors seem to play a minor role so far, which can only be indirectly deduced from our study. At least for now, drug availability is not a major concern as indicated by our participants. Fear of overdose was prevalent in only 13% of patients in our sample and even dropped to 6% since the onset of COVID-19. This lack of awareness of possible overdoses in our sample is also a cause for concern, as the majority of our participants usually consumed alone, even before the onset of the pandemic. This bears the danger that no help can be administered in case of an overdose as discussed earlier (7). Crucially, our observations are restricted to patients in treatment. We can therefore not assure that drug users, who are not seeking help, might be at greater risk of overdose during the pandemic. Therefore, emphasizing the increase of potential overdoses for persons who use drugs due to many factors brought along by COVID-19 should be implemented in current health care strategies.

## Discussion

At the initial stage of the pandemic, the impact of the COVID-19 pandemic in terms of incidents and mortality has been less severe in Austria compared to other countries in Europe and worldwide. From our point of view as a clinical facility treating patients with drug use disorder, drug use behavior, and the drug market seemed also less directly affected by COVID-19 than anticipated in Austria, at least at the initial stage of the pandemic. This is also reflected in our data collected from a small clinical population of patients with a high level of drug dependency. Although this group clearly indicated an impact of the pandemic on many aspects of their personal life, individual drug use patterns seemed less affected at this initial stage. Furthermore, the Austrian drug market in terms of pricing and availability appeared also rather stable, which is in line with other expert opinions and our overall observations at our clinical facility. The overall maintenance of the Austrian health system due to the less severe impact of COVID-19 so far could be hypothesized as possible reasons for the stable drug situation.

We urge to not misinterpret this surprising lack of direct massive impact of COVID-19 on this clinical group as an all-clear. In fact, close monitoring of the development of this clinical population is of great importance, since long-term effects have yet to be investigated. For instance, the already difficult job situation for patients struggling with addiction might result in long-term negative consequences given the general increase in unemployment due to COVID-19 in the general population. Furthermore, existing psychological problems might deteriorate resulting in higher numbers of comorbidities and co-addictions. Finally, pushing this clinical group further to the edge of society can have severe consequences for their well-being. In this still ongoing pandemic it cannot be foreseen, when the impact on this already deprived population struggling with many problems reaches its peak and the situation starts getting out of control. Therefore, stability in access to addiction treatment should be emphasized with regard to the COVID-19 pandemic and resulting government measures.

Prevalent misuse and concomitant use in OST are particularly alarming and need to be addressed rapidly, while maintaining a high standard in care. This is especially challenging during the COVID-19 pandemic, with many resources of the health care system fully occupied with controlling the disease and its impact on other mental health issues. With COVID-19 on the rise again and the multiple known risk factors for people with drug addiction, development of long-term strategies to improve the outlook for this vulnerable group cannot be neglected.

In conclusion, the immediate impact of COVID-19 on highly addicted patients with drug use disorder in treatment, was less severe than expected. We emphasize, that our perspectives are based on observations at a clinical facility and restricted to the described clinical sample. As a major health care provider in our region (Upper Austria), a wide range of consequences on our patients can directly be observed and developments on the Austrian drug market can be deflected from our patients' reports. However, we emphasize that no direct conclusions for the general population can be drawn from our impressions and our small sample.

## Data Availability Statement

The raw data supporting the conclusions of this article will be made available by the authors, without undue reservation.

## Ethics Statement

The studies involving human participants were reviewed and approved by Ethics Commission of the Medical Faculty of the Johannes Kepler University Linz. Written informed consent for participation was not required for this study in accordance with the national legislation and the institutional requirements.

## Author Contributions

IF-L: conceptualization, formal analysis, methodology, and writing—original draft preparation. KY: supervision, data curation, resources, and writing—review. NG: conceptualization, data curation, and writing—review. JR: conceptualization, data curation, data preparation, and writing—review & editing. All authors: contributed to and have approved the final manuscript.

## Conflict of Interest

The authors declare that the research was conducted in the absence of any commercial or financial relationships that could be construed as a potential conflict of interest.

## Footnotes

^1^Sources: www.data.gv.at/covid-19; www.acaps.org

## References

[1] Rajkumar RP. COVID-19 and mental health: a review of the existing literature. Asian J Psychiatr. (2020) 52:102066. 10.1016/j.ajp.2020.102066 32302935 PMC7151415

[2] Dubey MJ Ghosh R Chatterjee S Biswas P Chatterjee S Dubey S. COVID-19 and addiction. Diabetes Metab Syndr. (2020) 14:817–23. 10.1016/j.dsx.2020.06.008 32540735 PMC7282772

[3] Jemberie WB Stewart Williams J Eriksson M Grönlund A-S Ng N Blom Nilsson M. Substance use disorders and COVID-19: multi-faceted problems which require multi-pronged solutions. Front. Psychiatry. (2020) 11:714. 10.3389/fpsyt.2020.00714 32848907 PMC7396653

[4] Columb D Hussain R O'Gara C. Addiction psychiatry and COVID-19: impact on patients and service provision. Ir J Psychol Med. (2020) 37:164–8. 10.1017/ipm.2020.47 32434597 PMC7280151

[5] Khatri UG Perrone J. Opioid use disorder and COVID-19: crashing of the crises. J Addict Med. (2020) 14:e6–7. 10.1097/ADM.0000000000000684 32404651 PMC7236857

[6] Saeedi M Omrani-Nava V Maleki I Hedayatizadeh-Omran A Ahmadi A Moosazadeh M Opium addiction and COVID-19: truth or false beliefs. Iran J Psychiatr Behav Sci. (2020) 14:e103509 10.5812/ijpbs.103509

[7] Volkow ND. Collision of the COVID-19 and addiction epidemics. Ann Intern Med. (2020) 173:61–2. 10.7326/M20-1212 32240293 PMC7138334

[8] Dietze PM Peacock A. Illicit drug use and harms in Australia in the context of COVID-19 and associated restrictions: Anticipated consequences and initial responses. Drug Alcohol Rev. (2020) 39:297–300. 10.1111/dar.13079 32445265 PMC7283853

[9] Farhoudian A Baldacchino AM Clark N Gerra G Ekhtiari H Dom G COVID-19 and substance use disorders: recommendations to a comprehensive healthcare response. An International Society of Addiction Medicine (ISAM) practice and policy interest group position paper. BCN. (2020) 11:133–46. 10.32598/bcn.11.covid19.1 32855772 PMC7368103

[10] Marsden J Darke S Hall W Hickman M Holmes J Humphreys K. Mitigating and learning from the impact of COVID-19 infection on addictive disorders. Addiction. (2020) 115:1007–10. 10.1111/add.15080 32250482 PMC9364227

[11] Mackolil J Mackolil J. Addressing psychosocial problems associated with the COVID-19 lockdown. Asian J Psychiatr. (2020) 51:102156. 10.1016/j.ajp.2020.102156 32413617 PMC7207101

[12] Wakeman SE Green TC Rich J. An overdose surge will compound the COVID-19 pandemic if urgent action is not taken. Nat Med. (2020) 26:819–20. 10.1038/s41591-020-0898-0 32555514 PMC8600654

[13] Dunlop A Lokuge B Masters D Sequeira M Saul P Dunlop G. Challenges in maintaining treatment services for people who use drugs during the COVID-19 pandemic. Harm Reduct J. (2020) 17:26. 10.1186/s12954-020-00370-7 32375887 PMC7201394

[14] DeJong CAJ DeJong-Verhagen JG Pols R Verbrugge CAG Baldacchino A. Psychological impact of the acute COVID-19 period on patients with substance use disorders: we are all in this together. Basic Clin. Neurosci. J. (2020) 11:207–16. 10.32598/bcn.11.covid19.2543.1 32855780 PMC7368105

[15] Martinotti G Alessi MC Di Natale C Sociali A Ceci F Lucidi L. Psychopathological burden and quality of life in substance users during the COVID-19 lockdown period in Italy. Front Psychiatry. (2020) 11:572245. 10.3389/fpsyt.2020.572245 33101086 PMC7497905

[16] Dale-Perera A Goulao J Stöver H Quality of care provided to patients receiving Opioid maintenance treatment in Europe: results from the EQUATOR analysis. Heroin Addict Relat Clin Prob. (2012) 14:23–38.

[17] Reimer J Wright N Somaini L Roncero C Maremmani I McKeganey N. The impact of misuse and diversion of opioid substitution treatment medicines: evidence review and expert consensus. Eur Addict Res. (2016) 22:99–106. 10.1159/000438988 26426530

[18] Gibney E. Whose coronavirus strategy worked best? Scientists hunt most effective policies. Nature. (2020) 581:15–6. 10.1038/d41586-020-01248-1 32341558

[19] Berman AH Bergman H Palmstierna T Schlyter F. Evaluation of the Drug Use Disorders Identification Test (DUDIT) in criminal justice and detoxification settings and in a swedish population sample. Eur Addict Res. (2005) 11:22–31. 10.1159/000081413 15608468

[20] Sinadinovic K Wennberg P Berman AH. Internet-based screening and brief intervention for illicit drug users: a randomized controlled trial with 12-month follow-up. J Stud Alcohol Drugs. (2014) 75:313–8. 10.15288/jsad.2014.75.313 24650825

[21] European Monitoring Centre for Drugs and Drug Addiction EMCDDA Update on Implications of COVID-19 for People Who Use Drugs (PWUD) and Drug Service Providers. (2020). Available online at: URL: https://www.emcdda.europa.eu/system/files/publications/12879/emcdda-covid-update-1-25.03.2020v2.pdf (accessed August 18, 2020).

[22] European Monitoring Centre for Drugs and Drug Addiction EU Drug Markets: Impact of COVID-19. (2020). Available online at: URL: https://www.emcdda.europa.eu/system/files/publications/13097/EU-Drug-Markets_Covid19-impact_final.pdf.

[23] European Monitoring Centre for Drugs and Drug Addiction Impact of COVID-19 on Patterns of Drug Use and Drug- Related Harms in Europe. (2020). Available online at: URL: https://www.emcdda.europa.eu/system/files/publications/13130/EMCDDA-Trendspotter-Covid-19-Wave-2_1.pdf (accessed August 18, 2020).

