# Supplementary material for: Developments in Drug Addiction During COVID-19—An Austrian Perspective Based on a Clinical Sample
Source: Front Psychiatry. 2020 Nov 27;11:602033. doi: 10.3389/fpsyt.2020.602033 (PMC7728611; doi:10.3389/fpsyt.2020.602033)
Supplement: Supplementary file 1 [file Data_Sheet_1.docx]

Supplementary Material

| **Drug / Substance** |  | **Total** | **Percent** | **Preferred Drug** | **Percent** |
| --- | --- | --- | --- | --- | --- |
| OST medication | prescribed | 24 | 75.0 | 14 | 43.8 |
|  | unprescribed | 6 | 18.8 | 1 | 3.1 |
| Benzodiazepine | prescribed | 14 | 43.8 |  |  |
|  | unprescribed | 13 | 40.6 |  |  |
| Cannabis |  | 18 | 56.3 | 6 | 18.8 |
| Heroin |  | 11 | 34.4 | 8 | 25 |
| Amphetamines |  | 10 | 31.3 | 1 | 3.1 |
| Methamphetamine |  | 7 | 21.9 |  |  |
| Cocaine |  | 7 | 21.9 |  |  |
| Other medication or substances |  | 11 | 31.3 | 2 | 6.2 |

**Supplementary Table 1**. Consumed and preferred drugs and substances, displayed as total numbers and percentage values in respect to the total sample (N=32). Total numbers of referenced drugs as ‘preferred drug” and respective percentages are displayed in the right outer columns. For opioid substitution treatment (OST) medication and benzodiazepine a distinction between prescribed and unprescribed use is displayed. Among ‘other medication or substances’ ketamine, pregabalin, dihydrocodeine and morphine were mentioned.

| **Question / Drug Consumption Aspect** | | **Before COVID-19** | | **After COVID-19** | |
| --- | --- | --- | --- | --- | --- |
|  |  | **Total** | **Percent** | **Total** | **Percent** |
| Drug supply | Active (pickup)  Passive (delivery)  Both  n.a. | 29  0  2  1 | 90.6  0  6.3  3.1 | 28  0  2  2 | 87.5  0  6.3  6.3 |
| Consumption space | Public  Private  Both | 1  24  7 | 3.1  75.0  21.9 | 1  25  6 | 3.1  78.1  18.8 |
| Consumption pattern | Alone only  With another person  Small group | 17  10  5 | 53.1  31.3  15.6 | 22  9  1 | 68.8  28.1  3.1 |
| Pricing preferred drug | Prescription drugs  n.a.  Increase  Decrease  Unchanged | 9  3 | 28.1  9.4 | 4  1  15 | 12.5  3.1  46.9 |
| Fear of overdose | Yes  No  n.a. | 4  27  1 | 12.5  84.4  3.1 | 2  29  1 | 6.3  90.6  3.1 |
| Stockpiling | Yes  No  n.a. | 6  25  1 | 18.8  78.1  3.1 | 5  26  1 | 15.6  81.3  3.1 |
| Availability problems | Yes  No  n.a. | 3  27  2 | 9.4  84.4  6.2 | 7  23  2 | 21.9  71.9  6.2 |
| Quality | Range 1-5 | Mean value = 4.1 | | Mean value = 4.0 | |

**Supplementary Table 2**. Various aspects of drug consumption behavior with responses displayed as total numbers and percentages of participants in respect to the total sample (N=32).

| **Concomitant Used Drug** |  | **Total** | **Percent** |
| --- | --- | --- | --- |
| OST medication | unprescribed | 4 | 16.7 |
| Benzodiazepine | prescribed | 12 | 50.0 |
|  | unprescribed | 8 | 33.3 |
| Cannabis |  | 12 | 50.0 |
| Heroin |  | 7 | 29.2 |
| Amphetamines |  | 7 | 29.2 |
| Methamphetamine |  | 5 | 20.8 |
| Cocaine |  | 5 | 20.8 |
| Other medication or substances |  | 7 | 29.2 |

**Supplementary Table 3**. Concomitant consumed drugs and substances of people in opioid substitution treatment (OST), total numbers and percentages (N=24).
